# Basic reproduction number and predicted trends of coronavirus disease 2019 epidemic in the mainland of China

**DOI:** 10.1186/s40249-020-00704-4

**Published:** 2020-07-16

**Authors:** Yong Li, Lian-Wen Wang, Zhi-Hang Peng, Hong-Bing Shen

**Affiliations:** 1 School of Information and Mathematics, Yangtze University, Jingzhou, 434023 China; 2 Department of Mathematics, Hubei Minzu University, Enshi, 445000 China; 3 Department of Epidemiology and Biostatistics, School of Public Health, Nanjing Medical University, Nanjing, 210029 China

**Keywords:** Coronavirus disease 2019, SEIQR model, Basic reproduction number, Parameter estimation, Lockdown, Large-scale case-screening

## Abstract

**Background:**

Coronavirus disease 2019 (COVID-19) has caused a serious epidemic around the world, but it has been effectively controlled in the mainland of China. The Chinese government limited the migration of people almost from all walks of life. Medical workers have rushed into Hubei province to fight against the epidemic. Any activity that can increase infection is prohibited. The aim of this study was to confirm that timely lockdown, large-scale case-screening and other control measures proposed by the Chinese government were effective to contain the spread of the virus in the mainland of China.

**Methods:**

Based on disease transmission-related parameters, this study was designed to predict the trend of COVID-19 epidemic in the mainland of China and provide theoretical basis for current prevention and control. An SEIQR epidemiological model incorporating asymptomatic transmission, short term immunity and imperfect isolation was constructed to evaluate the transmission dynamics of COVID-19 inside and outside of Hubei province. With COVID-19 cases confirmed by the National Health Commission (NHC), the optimal parameters of the model were set by calculating the minimum Chi-square value.

**Results:**

Before the migration to and from Wuhan was cut off, the basic reproduction number in China was 5.6015. From 23 January to 26 January 2020, the basic reproduction number in China was 6.6037. From 27 January to 11 February 2020, the basic reproduction number outside Hubei province dropped below 1, but that in Hubei province remained 3.7732. Because of stricter controlling measures, especially after the initiation of the large-scale case-screening, the epidemic rampancy in Hubei has also been contained. The average basic reproduction number in Hubei province was 3.4094 as of 25 February 2020. We estimated the cumulative number of confirmed cases nationwide was 82 186, and 69 230 in Hubei province on 9 April 2020.

**Conclusions:**

The lockdown of Hubei province significantly reduced the basic reproduction number. The large-scale case-screening also showed the effectiveness in the epidemic control. This study provided experiences that could be replicated in other countries suffering from the epidemic. Although the epidemic is subsiding in China, the controlling efforts should not be terminated before May.

## Background

Coronavirus disease 2019 (COVID-19) is caused by a novel coronavirus, formerly named as 2019-nCoV by World Health Organization (WHO) on 12 January 2020 and then severe acute respiratory syndrome coronavirus 2 (SARS-CoV-2) by the International Committee on Taxonomy of Viruses (ICTV) on 11 February 2020 [1, 2]. The epidemic has spread rapidly across the world [3–5]. In China, a cluster of pneumonia cases was reported in Wuhan, Hubei province, in December 2019 [6–8]. Rapidly, Hubei province became the hardest-hit of the COVID-19 epidemic, due to the high rate of human-to-human transmission [9–11], mainly through droplets from coughing or sneezing or body contact. Immediately, the Chinese government took drastic controlling efforts (like public education, active surveillance, early detection, case management, contact tracing, especially mandatory quarantine for at least 14 days [12]). As of 26 May 2020 (24:00 GMT + 8), 82 993 confirmed cases, 6 suspected cases and 4634 deaths were reported in China, most of them from Hubei province (68 135 confirmed cases and 4512 deaths) [13]. This epidemic is attacking 215 countries and regions around the world, such as USA, Spain, Italy, the United Kingdom, Russian Federation, Germany, Brazil, France, Turkey, Iran, Canada, Peru, India, Belgium, Netherlands and Republic of Korea. There have appeared 5 404 512 confirmed cases and 343 514 deaths worldwide until 26 May 2020 (10:00 GMT + 2) [14].

SARS-CoV-2, the Middle East respiratory syndrome coronavirus (MERS-CoV), and the flu virus belong to the same family and cause similar symptoms [15, 16]. People of all ages are susceptible. Onset manifestations of COVID-19 include fever, cough symptoms, myalgia or fatigue, and a few or few present sputum overproduction, headache, haemoptysis and diarrhea [17–22]. Though showing no symptoms or only mild symptoms, some patients still transmit the virus, a phenomenon that is called asymptomatic transmission [23–26]. No specific treatment drugs have yet been created for COVID-19 [12, 27]. COVID-19 is self-limiting, and patients often heal through their immunity. Many asymptomatic patients can recover after self-quarantine [28].

The 2002–2003 SARS epidemic led to 8096 cases and 774 deaths (mortality 9.6%) in 29 countries or regions [29], and the persistent MERS epidemics led to 2494 cases and 858 deaths (mortality 34.4%) in 27 countries during 2012–2019 [30]. The COVID-19 epidemic has aroused global health concern [31]. Many countries implemented mandatory quarantine in spite of its relatively low mortality (2.8%). For instance, the government of China limited inflow and outflow of people almost from all walks of life, and suspended all kinds of mass activities. But, medical workers have streamed into Hubei province to fight against the epidemic.

Therefore, to find more effective control efforts, the peak arrival time and the trend of the COVID-19 epidemic in the mainland of China should be predicted with a well-designed model [32, 33]. Many studies have estimated the reproduction number in the early phase of COVID-19 outbreak in China [32–43].

This study based on the cumulative confirmed cases, cured and discharged cases, death tolls and suspected cases released from the National Health Commission (NHC) [13]. These findings formulate a SEIQR (susceptible-exposed-infected but not hospitalized-infectious and isolated-recovered) epidemic model to explore the impacts of the lockdown of Wuhan and the curb of population migration on COVID-19 transmission. The model was constructed, incorporating asymptomatic transmission, short term immunity and imperfect isolation. The aim of this study was to prove that timely lockdown, large-scale case-screening and other control measures proposed by the Chinese government were effective means to contain the spread of the virus in the mainland of China. Through parameter estimation, we got the parameters of the model. With the aid of sensitivity analysis, we evaluated the timeliness and correctness of the early measures implemented by the Chinese government. Before 25 February 2020, we predicted the epidemic trend in China, and the final number of cumulative confirmed cases in Hubei province and the mainland of China. Up to now, the results have been proved accurate. At the same time, the basic reproduction number of each stage is decreasing, which reflects that the control measures of the Chinese government play a decisive role. Before 25 February, we also gave suggestions on the time for full resumption of work. Although the epidemic is under control, it is not the time to terminate the controlling efforts which are expected to be ended as early as in May.

## Methods

### The COVID-19 model

In the mainland of China, the government of China is rigorously limiting the migration of people among all provinces, with Hubei province completely cut off from the outside. For this reason, Hubei province and other provinces are considered into two patches, denoted as 1 and 2, respectively (e.g., seeing [32, 44]). In each patch, the population related to COVID-19 is divided into five epidemiological subgroups: susceptible, *S*_*i*_; exposed, *E*_*i*_; infected but not hospitalized, *I*_*i*_ (including suspected, carrier and undetected); infectious and isolated (daily confirmed real-time cases announced by NHC [13]), *Q*_*i*_; and recovered (short term immunity), *R*_*i*_. The total population *N*_*i*_ = *S*_*i*_ + *E*_*i*_ + *I*_*i*_ + *Q*_*i*_ + *R*_*i*_, *i* = 1,2. In order to better reflect the actual situation of COVID-19 transmission in the mainland of China, we set the following conditions:

1. Natural birth and death are ignored since we only focus on the short-term disease transmission;
2. Asymptomatic transmission [23–26] is the one mode of transmission of COVID-19; the susceptible (*S*_*i*_) individuals may be infected due to contacts with the infected but not hospitalized individuals (*I*_*i*_). The individuals in incubation period (*E*_*i*_) also have the potential to transmit the virus. Meanwhile, infectious and isolated individuals (*Q*_*i*_) also have a certain probability to transmit the virus to medical workers and others, which is a phenomenon called imperfect isolation. Therefore, the exposed (*E*_*i*_) and the infectious and isolated (*Q*_*i*_) are considered infectious, with infectivity reduction factors *k*_*i*_ and *l*_*i*_, respectively;
3. A few infected individuals do not develop obvious symptoms and have no short-term immunity after self-healing [28], so it is assumed that these individuals (*γ*_*i*_*η*_*i*_*I*_*i*_) will directly return to the susceptible;
4. Wuhan was locked down off at 10:00 AM, 23 January 2020, and other cities in Hubei province were locked down successively. Xiangyang City in Hubei province was lastly locked down at 00:00 on 27 January 2020. So, the migration rates are considered as follows:

$$ \omega =\left\{\begin{array}{l}{\omega}_a,11-22\ \mathrm{January}\ 2020,\mathrm{Wuhan}\ \mathrm{was}\ \mathrm{not}\ \mathrm{locked}\ \mathrm{down},\\ {}{\omega}_b,23-26\ \mathrm{January}\ 2020,\mathrm{Wuhan}\ \mathrm{was}\ \mathrm{locked}\ \mathrm{down},\\ {}{\omega}_c,\mathrm{After}\ 26\ \mathrm{January}\ 2020,\mathrm{Hubei}\ \mathrm{was}\ \mathrm{locked}\ \mathrm{down}.\end{array}\right. $$

5) The lockdown of Wuhan city and Hubei province, and the large-scale case-screening starting on 12 February may bring out impacts on the transmission rates (*β*_*i*_*i* = 1,2) among individuals, so

$$ {\beta}_{\mathrm{i}}=\left\{\begin{array}{l}{\beta}_{ia},11-22\ \mathrm{January}\ 2020,\mathrm{Wuhan}\ \mathrm{was}\ \mathrm{not}\ \mathrm{locked}\ \mathrm{down},\\ {}{\beta}_{ib},23-26\ \mathrm{January}\ 2020,\mathrm{Wuhan}\ \mathrm{was}\ \mathrm{locked}\ \mathrm{down},\\ {}{\beta}_{ic},26\ \mathrm{January}-11\ \mathrm{February}\ 2020,\mathrm{Hubei}\ \mathrm{was}\ \mathrm{locked}\ \mathrm{down},\\ {}{\beta}_{id},\mathrm{After}\ 11\ \mathrm{February}\ 2020,\mathrm{the}\ \mathrm{large}\hbox{-} \mathrm{scale}\ \mathrm{case}\hbox{-} \mathrm{screening}\ \mathrm{has}\ \mathrm{begun}.\end{array}\right. $$

6) Before the lockdown of Hubei province, we consider that infectivity reduction factors (*l*_*i*_, *i* = 1,2) between medical workers and patients before 26 January is much higher than that after 26 January, due to the shortage of medical resources and the insufficient understanding of COVID-19 transmission:

$$ {l}_i=\left\{\begin{array}{l}{l}_a,11-22\ \mathrm{January}\ 2020,\mathrm{before}\ \mathrm{conditions}\ \mathrm{for}\ \mathrm{medical}\ \mathrm{staff}\ \mathrm{was}\ \mathrm{improved},\\ {}{l}_b,23-26\ \mathrm{January}\ 2020,\mathrm{after}\ \mathrm{conditions}\ \mathrm{for}\ \mathrm{medical}\ \mathrm{staff}\ \mathrm{was}\ \mathrm{improved},\\ {}{l}_c,26\ \mathrm{January}-11\ \mathrm{February},2020,\mathrm{medical}\ \mathrm{staff}\ \mathrm{was}\ \mathrm{well}\ \mathrm{protected},\\ {}{l}_d,\mathrm{After}\ 11\ \mathrm{February}\ 2020,\mathrm{the}\ \mathrm{protection}\ \mathrm{of}\ \mathrm{medical}\ \mathrm{staff}\ \mathrm{has}\ \mathrm{been}\ \mathrm{further}\ \mathrm{improved}.\end{array}\right. $$

**Fig. 1:**
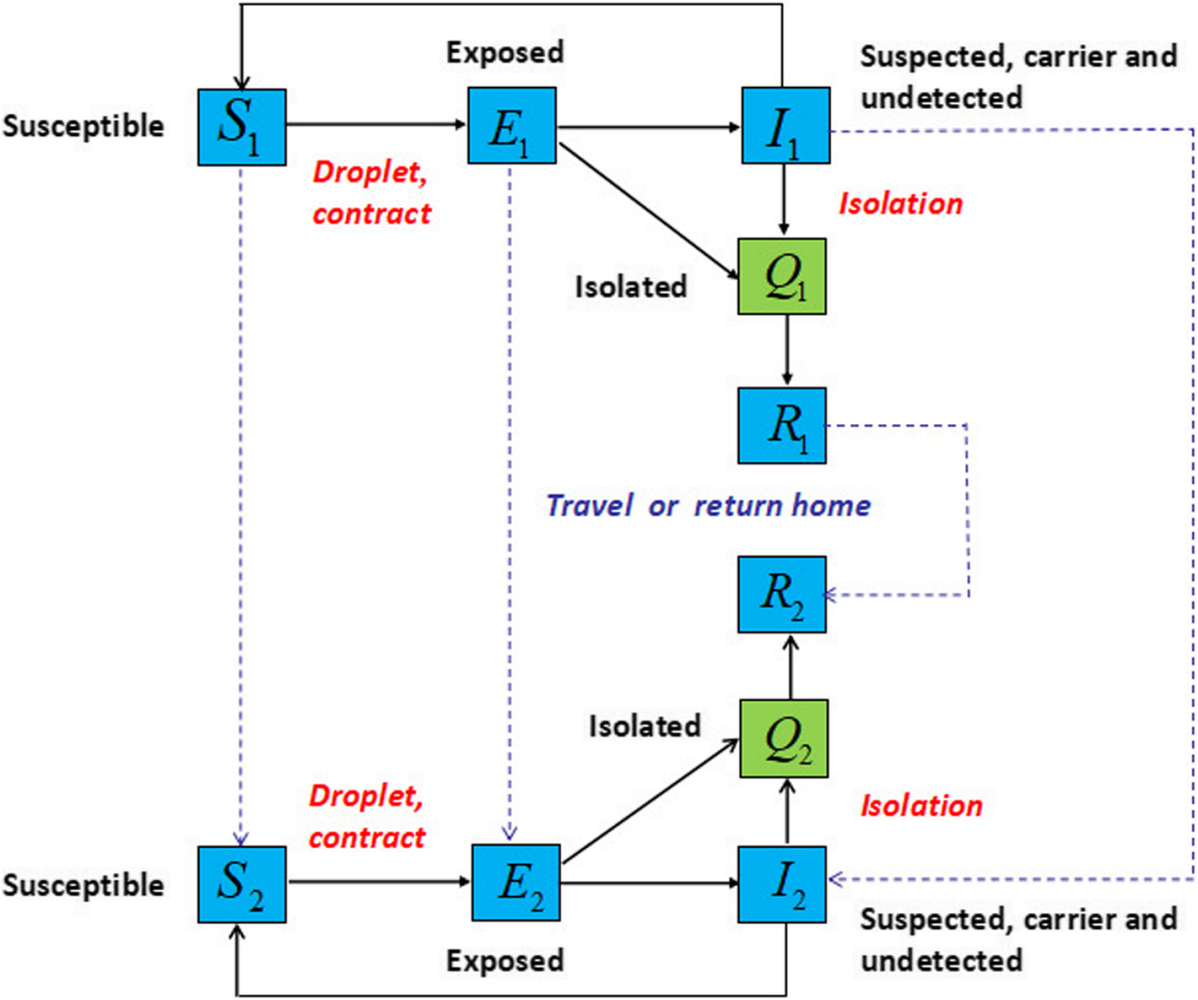
Flow chart of compartments of the COVID-19 SEIQR model

A schematic flow diagram was created for illustrating the transmission dynamics of the COVID-19 infection in Fig. 1. And the biological meanings and acceptable ranges of all parameters are listed in Table 1. The model is described by the following system of ordinary differential equations:

1 $$ \left\{\begin{array}{l}\frac{dS_1}{dt}=-{\beta}_1{S}_1\frac{\left({I}_1+{k}_1{E}_1+{l}_1{Q}_1\right)}{N_1}-\omega {S}_1+{\gamma}_1{\eta}_1{I}_1,\\ {}\frac{dE_1}{dt}={\beta}_1{S}_1\frac{\left({I}_1+{k}_1{E}_1+{l}_1{Q}_1\right)}{N_1}-\left({\alpha}_1+\omega \right){E}_1,\\ {}\frac{dI_1}{dt}={\alpha}_1\left(1-{\rho}_1\right){E}_1-\left({\gamma}_1+\omega \right){I}_1,\\ {}\frac{dQ_1}{dt}={\alpha}_1{\rho}_1{E}_1+{\gamma}_1\left(1-{\eta}_1\right){I}_1-\left({\delta}_1+d\right){Q}_1,\\ {}\frac{dR_1}{dt}={\delta}_1{Q}_1-\omega {R}_1,\\ {}\frac{dS_2}{dt}=-{\beta}_2{S}_2\frac{\left({I}_2+{k}_2{E}_2+{l}_2{Q}_2\right)}{N_2}+\omega {S}_1+{\gamma}_2{\eta}_2{I}_2,\\ {}\frac{dE_2}{dt}={\beta}_2{S}_2\frac{\left({I}_2+{k}_2{E}_2+{l}_2{Q}_2\right)}{N_2}-{\alpha}_2{E}_2+\omega {E}_1,\\ {}\frac{dI_2}{dt}={\alpha}_2\left(1-{\rho}_2\right){E}_2-{\gamma}_2{I}_2+\omega {I}_1,\\ {}\frac{dQ_2}{dt}={\alpha}_2{\rho}_2{E}_2+{\gamma}_2\left(1-{\eta}_2\right){I}_2-\left({\delta}_2+d\right){Q}_2,\\ {}\frac{dR_2}{dt}={\delta}_2{Q}_2+\omega {R}_1.\end{array}\right. $$

**Table 1:** Parameter estimates for COVID-19 in the mainland of China

Parameter, initial value	Definition	Value	Standard deviation	Source
*β*_1*a*_ ∈ [0.01, 10]	Transmission rate (day^− 1^ individual^− 1^)	1.0009	0.1945	Estimated
*β*_1*b*_ ∈ [0.01, 10]	Transmission rate	1.3754	1.076	Estimated
*β*_1*c*_ ∈ [0.01, 10]	Transmission rate	1.0967	0.0501	Estimated
*β*_1*d*_ ∈ [0.01, 10]	Transmission rate	0.0809	0.0371	Estimated
*β*_2*a*_ ∈ [0.01, 10]	Transmission rate	2.2783	1.0798	Estimated
*β*_2*b*_ ∈ [0.01, 10]	Transmission rate	1.4721	0.6584	Estimated
*β*_2*c*_ ∈ [0.01, 10]	Transmission rate	0.289	0.0176	Estimated
*β*_2*d*_ ∈ [0.01, 10]	Transmission rate	0.0189	0.0091	Estimated
*δ*_1_ = *δ*_2_ ∈ [0.0333, 0.1]	Recovery rate (day^− 1^)	0.0618	0.0094	Estimated
*γ*_1_ = *γ*_2_ ∈ [0.1, 0.3333]	Detection rate (day^− 1^)	0.2269	0.0457	Estimated
*ω*_*a*_ ∈ [0.0027, 0.03]	Migration rate (day^− 1^)	0.0033	0.0002	Estimated
*ω*_*b*_ ∈ [0.0027, 0.03]	Migration rate (day^− 1^)	0.0029	0.0006	Estimated
*l*_*a*_ ∈ [0.1, 1]	Infectivity reduction factor	0.2001	0.0152	Estimated
*l*_*b*_ ∈ [0.1, 1]	Infectivity reduction factor	0.1489	0.0164	Estimated
*l*_*c*_ ∈ [0.1, 1]	Infectivity reduction factor	0.0587	0.0159	Estimated
*l*_*d*_	Infectivity reduction factor	0.0001	–	Fixed
*η*_1_ = *η*_2_ ∈ [0.01, 0.1]	Self-healing ratio	0.0335	0.0056	Estimated
*k* ∈ [0.1, 1]	Infectivity reduction factor (day^− 1^)	0.3301	0.0678	Estimated
*α*_1_ = *α*_2_	Transition rate of exposed (day^− 1^)	0.1724	–	[45]
*ρ*_1_ = *ρ*_2_	Proportion of the infectious	0.8683	–	[33]
*d*	Disease-induced death rate (day^− 1^)	1.7826 × 10 ^− 5^	–	[33]
*S*_1_ (0)	Initial susceptible population	1.10 × 10 ^7^	–	[46, 47]
*E*_1_ (0) ∈ [0, 10 ^5^]	Initial exposed population	12.2544	4.3719	Estimated
*I*_1_ (0) ∈ [0, 10 ^5^]	Initial infected population	0.1208	0.0868	Estimated
*Q*_1_ (0)	Initial isolated population	41	–	Data
*R*_1_ (0)	Initial recovered population	2	–	Data
*S*_2_ (0) ∈ [10 ^3^,1.3 × 10 ^9^]	Initial susceptible population	1.23 × 10 ^8^	1.31 × 10 ^5^	Estimated
*E*_2_ (0) ∈ [0,10 ^4^]	Initial exposed population	0.0184	0.0094	Estimated
*I*_2_ (0) ∈ [0,10 ^5^]	Initial infected population	0.0154	0.0087	Estimated
*Q*_2_ (0)	Initial isolated population	0	–	Data
*R*_2_ (0)	Initial recovered population	0	–	Data

**-** means not applicable

### The basic reproduction number (*R*_0_)

The basic reproduction number (*R*_0_) represents the number of infected during the patient’s early infectious period (asymptomatic). This threshold may determine whether a disease will die out (if *R*_0_ < 1) or become epidemic (if *R*_0_ > 1). As far as the epidemic demonstrates complex dynamics, *R*_0_ < 1 is not only the condition guaranteeing that the fate of the disease, but the smaller the better. Following Driessche and Watmough [48], we can compute the basic reproduction number as *R*_0_ = max {*R*_0_^(1)^, *R*_0_^(2)^} before 27 January 2020:

$$ {\displaystyle \begin{array}{l}{R_0}^{(1)}=\underset{\mathrm{contact}\ \mathrm{with}\ \mathrm{exposed}\ \mathrm{class}}{\underbrace{\frac{\beta_1{k}_1}{\alpha_1+\omega }}}+\underset{\mathrm{contact}\ \mathrm{with}\ \mathrm{infected}\ \mathrm{class}}{\underbrace{\frac{\beta_1{\alpha}_1\left(1-{\rho}_1\right)}{\left({\alpha}_1+\omega \right)\left({\gamma}_1+\omega \right)}}}+\underset{\mathrm{contact}\ \mathrm{with}\ \mathrm{isolated}\ \mathrm{class}}{\underbrace{\frac{\beta_1{l}_1{\alpha}_1\left[{\rho}_1\left({\gamma}_1+\omega \right)+{\gamma}_1\left(1-{\rho}_1\right)\left(1-{\eta}_1\right)\right]}{\left({\alpha}_1+\omega \right)\left({\gamma}_1+\omega \right)\left({\delta}_1+d\right)}}}\\ {}\kern1.5em := {R}_{01}+{R}_{02}+{R}_{03}\end{array}} $$

$$ {\displaystyle \begin{array}{l}{R_0}^{(2)}=\underset{\mathrm{travel}\ \mathrm{with}\ \mathrm{exposed}\ \mathrm{class}}{\underbrace{\frac{{\omega \beta}_2{k}_2}{\alpha_2\left({\alpha}_1+\omega \right)}}}+\underset{\mathrm{travel}\ \mathrm{with}\ \mathrm{infected}\ \mathrm{class}}{\underbrace{\frac{{\omega \beta}_2\left[{\alpha}_1\left(1-{\rho}_1\right)+\left({\gamma}_1+\omega \right)\left(1-{\rho}_2\right)\right]}{\gamma_2\left({\alpha}_1+\omega \right)\left({\gamma}_1+\omega \right)}}}\\ {}+\underset{\mathrm{travel}\ \mathrm{with}\ \mathrm{isolated}\ \mathrm{class}}{\underbrace{\frac{{\omega \beta}_2{l}_2\left[{\alpha}_1\left(1-{\rho}_1\right)\left(1-{\eta}_2\right)+\left({\gamma}_1+\omega \right)\left(1-{\rho}_2\right)\left(1-{\eta}_2\right)+{\rho}_2\left({\gamma}_1+\omega \right)\right]}{\left({\alpha}_1+\omega \right)\left({\gamma}_1+\omega \right)\left({\delta}_1+d\right)\left({\delta}_2+d\right)}}}\\ {}\kern1.5em := {R}_{04}+{R}_{05}+{R}_{06}\end{array}} $$

Here, *R*_01_, *R*_02_ and *R*_03_ represent the average numbers of the infected individuals by a single exposed individual (*E*_1_), infected but not hospitalized individual (*I*_1_) or infectious and isolated individual (*Q*_1_) in a fully susceptible population, respectively. *R*_04_, *R*_05_ and *R*_06_ represent the average numbers of the infected individuals by a single exposed individual (*E*_1_ and *E*_2_), infected but not hospitalized individual (*I*_1_ and *I*_2_) or infectious and isolated individual (*Q*_1_ and *Q*_2_) in a fully susceptible population travelling to and for, respectively. They represent the contributions of six transmission ways of COVID-19 to the basic reproduction number *R*_0_.

After 26 January 2020, the whole Hubei province was cut off from the outside, then system (1) is transformed into two independent systems. The basic reproduction number of Hubei province (*R*_0_^(1)^) and that outside of Hubei (*R*_0_^(2)^) become

$$ {R}_0(i)=\frac{\beta_i{k}_i}{\alpha_i}+\frac{\beta_i\left(1-{\rho}_i\right)}{\gamma_i}+\frac{\beta_i{l}_i\left[\left(1-{\rho}_i\right)\left(1-{\eta}_i\right)+{\rho}_i\right]}{\delta_i+d},i=1,2. $$

## Parameters estimated

### Data source

The NHC releases daily reports on cumulative confirmed cases of COVID-19 (positive nucleic acid test result), cured and discharged cases, death tolls and suspected cases of COVID-19 in the mainland of China and Hubei province from 0 to 24 o’clock [13]. On 5 February 2020, NHC released the fifth edition of the *Diagnosis and Treatment Protocol for COVID-19* [49]. On 12 February 2020, NHC advocated the large-scale case-screening of previously suspected cases and reexamination of the diagnostic results, and that the diagnosis of COVID-19 should be based on three criteria (previously on two): a history of epidemiological contact or a history of stay in the epidemic area, clinical symptoms (such as fever, cough) and CT features. The NHC announced 59 804 confirmed cases nationwide, including 13 332 clinically diagnosed cases on 12 February. In the next 2 days, the number rose to 63 851 (15 384 clinically diagnosed cases) and 66 492 (16 522 clinically diagnosed cases). With the release of the sixth edition of the *Diagnosis and Treatment Protocol for COVID-19* [50], it was officially announced on February 19 that the original two standards were reused for the diagnosis.

By subtracting the data of Hubei province from the national data, we obtained the cumulative case data outside Hubei province. The study involved case data released from 11 January to 25 February 2020. We fitted the daily cumulative confirmed data in Hubei province and those outside Hubei province, and further predicted the number of final confirmed cases. The isolated *Q*_*i*_(*t*), *i* = 1, 2, in model (1) represented the daily real-time confirmed cases of Hubei province and outside of Hubei province, respectively. So, the following two equations could describe the dynamics of the cumulative confirmed cases (cumulative isolated) in Hubei province and outside Hubei province, respectively:

$$ \left\{\begin{array}{l}\frac{dL_1}{dt}={\alpha}_1{\rho}_1{E}_1+{\gamma}_1\left(1-{\eta}_1\right){I}_1,\\ {}\frac{dL_2}{dt}={\alpha}_2{\rho}_2{E}_2+{\gamma}_2\left(1-{\eta}_2\right){I}_2.\end{array}\right. $$

### Parameter estimation

From the work of Tang et al. [33], we set the proportion of the infectious *ρ*_1_ = *ρ*_2_ = 0.8683, disease-induced death rate *d* = 1.7826 × 10^− 5^. The susceptible population in Hubei province was considered as the permanent population in Wuhan city. According to the statistical yearbook data of Wuhan city [46, 47], we then assumed *S*_1_(0) = 1.1 × 10^7^. According to the cumulative daily case data reported from the NHC [13], we set *Q*_1_(0) = 41, *R*_1_(0) = 2, *Q*_2_(0) = 0, *R*_2_(0) = 0. The incubation period (1/*α*_*i*_, *i* = 1, 2) of COVID-19 was 5.8 days [45], so transition rate of exposed individuals *E*_*i*_ read *α*_1_ = *α*_2_= 0.1724. We consulted the values of *k* and *l* in the literature [51] and got the appropriate range of *k* and *l*. Considering that the patient’s disease course (1/*δ*_*i*_, *i* = 1, 2) is about 10 to 30 days, and the time required to detect a suspected patient (1/*γ*_*i*_, *i* = 1, 2) is 3 to 10 days, we thus set the ranges of parameters *δ*_*i*_ and *γ*_*i*_, *i* = 1, 2, respectively. Based on [34, 37], the range of transmission rate *β*_*i*_, *i* = 1, 2, was given. Before the lockdown of Wuhan, we assume that 30% (about 300 000) of the total population in Wuhan had traveled to and back home every day from 11 to 22 January. The average time to move out of Wuhan was 1 day. Therefore, the upper limit of the rate of migration out of Wuhan (all citizens moved out, *ω*) is 0.03 × 1/1 = 0.03 and the lower limit of the mobility is 1/365 = 0.0027, considering that everyone moves out of Wuhan at least once a year. The lower and upper limits of other parameters and initial values of model (1) are shown in Table 1.

The detailed steps of simulations were stated as follows.

1. **Before closing the city (11 January–22 January 2020)**: The period is the prophase of high-rate transmission since people do not know that COVID-19 can be transmitted from person to person;
2. **After Wuhan was locked down and before Hubei province was locked down (23 January–26 January 2020)**: Wuhan was locked down at 10:00 AM on 23 January, and the last city of Hubei province was locked down (Xiangyang city) in the early morning of 27 January. During this period, the lockdown may bring many sharp impacts. Therefore, the values of mobility rate (*ω*) and transmission rate (*β*_*i*_, *i* = 1, 2) vary, see Table 1 for details.
3. **After Hubei province was completely locked down, and before the complete case-screening mainly in Hubei province started (23 January–11 February 2020)**: On and after 27 January, all cities in Hubei province were gradually locked down. In this situation, there may be no migration between Hubei province and other provinces, so the migration rate could be fixed as 0. Supposing that the transmission rate (*β*_*i*_, *i* = 1, 2) and the infectivity reduction factors (*l*) between the medical staff and the patient vary. In addition, with the lockdown of all cities across the mainland of China, Hubei province implemented a more rigorous control. We believed that a large number of susceptible persons (*S*) were in a relatively safe situation and could not be infected. Therefore, on the 27th day, we assumed that the number of susceptible people (i.e., *S*_1_(17) and *S*_2_(17), 27 January was the 17th day of our simulation) changed greatly due to the lockdown of cities and traffic restrictions throughout the mainland of China, but not in other types of population (i.e., *E*, *I*, *Q* and *R*).
4. **After large-scale case-screening mainly in Hubei province started (after 11 February 2020)**: On 12 February, the cumulative confirmed cases announced by the NHC increased by 13 332 clinically diagnosed cases in Hubei province. At the same time, large-scale case-screening were carried out nationwide, and stricter control measures were implemented in Hubei province to further restrict residents’ move. Therefore, we must assess the impact of the large-scale case-screening that began on 12 February. For other provinces, using the model (1) to reflect the impact of the large-scale case-screening, we assumed that only the transmission rate (*β*_*i*_, *i* = 1, 2) was further reduced. Owing to the increasing medical supplies and deepening understanding of the virus, infection in doctors by patients at this time remained extremely rare, so the infectivity reduction factor (*l*) can be almost ignored (*l*_*d*_ = 0.0001) on 12 February (12 February was the 33th day of our simulation). Hence, we used the fewest parameters to characterize the impact of large-scale case-screening on the epidemic. For the confirmed cases of Hubei province on 12 February, it is equivalent to consider that the model has changed its dynamics on that day. Namely, except for the transmission rate (*β*_1_) and infectivity reduction factor (*l*) change, all variables of the model (1) of Hubei province have changed. The changes are analyzed in Section 5.

Hence, we segmented setting basis of parameters (mobility rate *ω*, transmission rate *β*_*i*_, *i* = 1, 2, and the infectivity reduction factors between the medical staff and the patient *l*) in Section 2. The last data in the four stage simulated was collected on 25 February 2020. From 11 January to 25 February 2020, there were 46 confirmed cases in Hubei province and 46 confirmed cases outside Hubei province.

As evidenced by the small cumulative number of confirmed cases in the prophase of the outbreak and large cumulative number of confirmed cases in the metaphase, the numeric curve fluctuates greatly. In order to achieve a better fitting effect, the Chi-square value was chosen to evaluate the reliability of model (1). We estimated the remaining 17 parameters and 5 initial values through calculating the minimum sum of Chi-square [52, 53].

$$ J=\sum \limits_{i=1}^{46}\frac{{\left(L\left({t}_i\right)-\hat{L}\left({t}_i\right)\right)}^2}{\hat{L}\left({t}_i\right)} $$

with the MATLAB (the Mathworks, Inc.). Here, *L*(*t*_*i*_), *i* = 1, 2, ⋯, 46 represent the actual daily confirmed cases, $\hat{L}\left({t}_i\right),i=1,2,\cdots, 46$ stand for the responding fitting values. The parameter values of model (1) at different stages and the initial values of model (1) on 11 January were given in Table 1.

## Results

### Fitting results and analysis of control measures

**Fig. 2:**
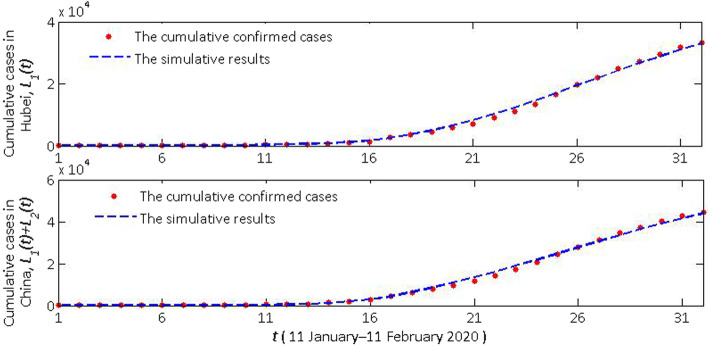
The cumulative daily confirmed and simulative cases in Hubei province (*L*_1_(*t*)) and the mainland of China (*L*_1_(*t*) + *L*_2_(*t*))

Cumulative daily cases (*L*_1_(t)) and actual confirmed cases in Hubei province and cumulative daily cases (*L*_1_(t) + *L*_2_(t)) and actual confirmed cases in the mainland of China are seen in Figs. 2, 4 and 5. According to the description in the previous section, the model (1) has changed its pattern for three times. The simulation results are very consistent with the actual cumulative confirmed cases. Next, we detailed the rationality of these major controlling measures adopted by NHC and the necessity of adapting our model to a new situation.

**Fig. 3:**
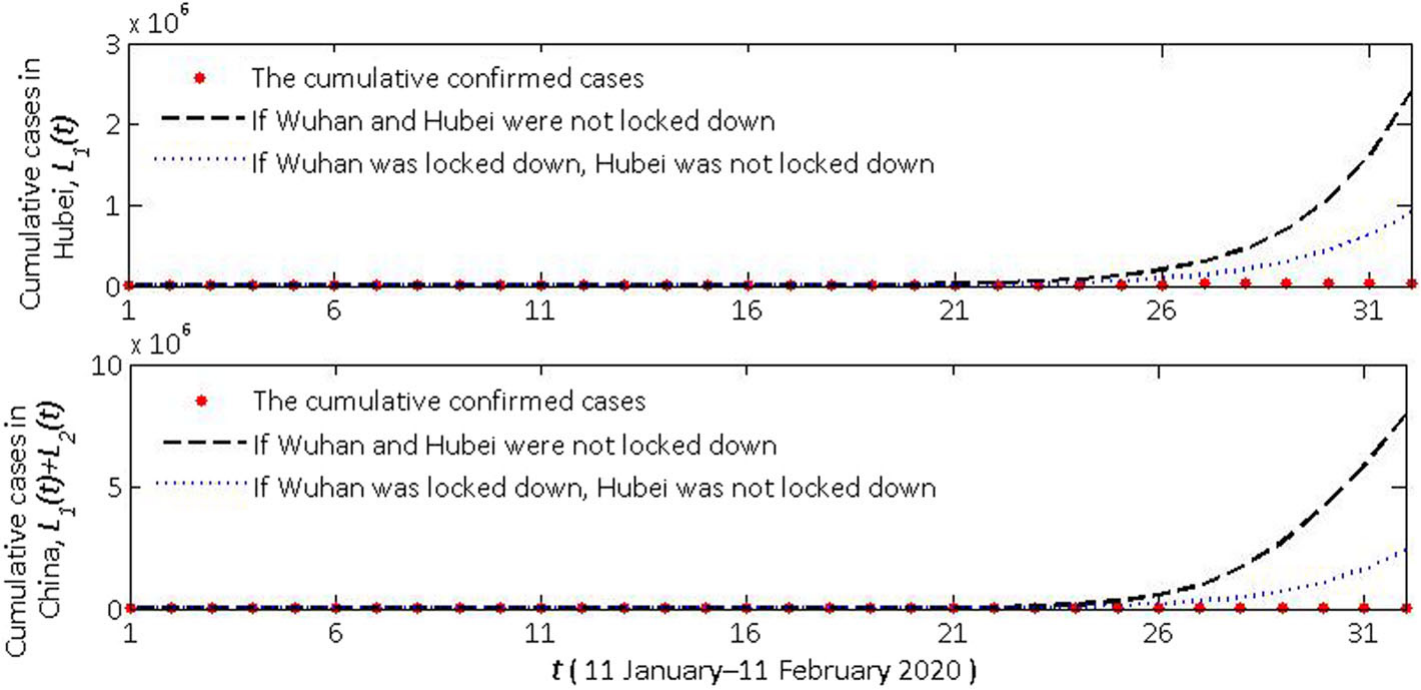
Comparison of the influence of lockdown of Wuhan and Hubei, China

**Fig. 4:**
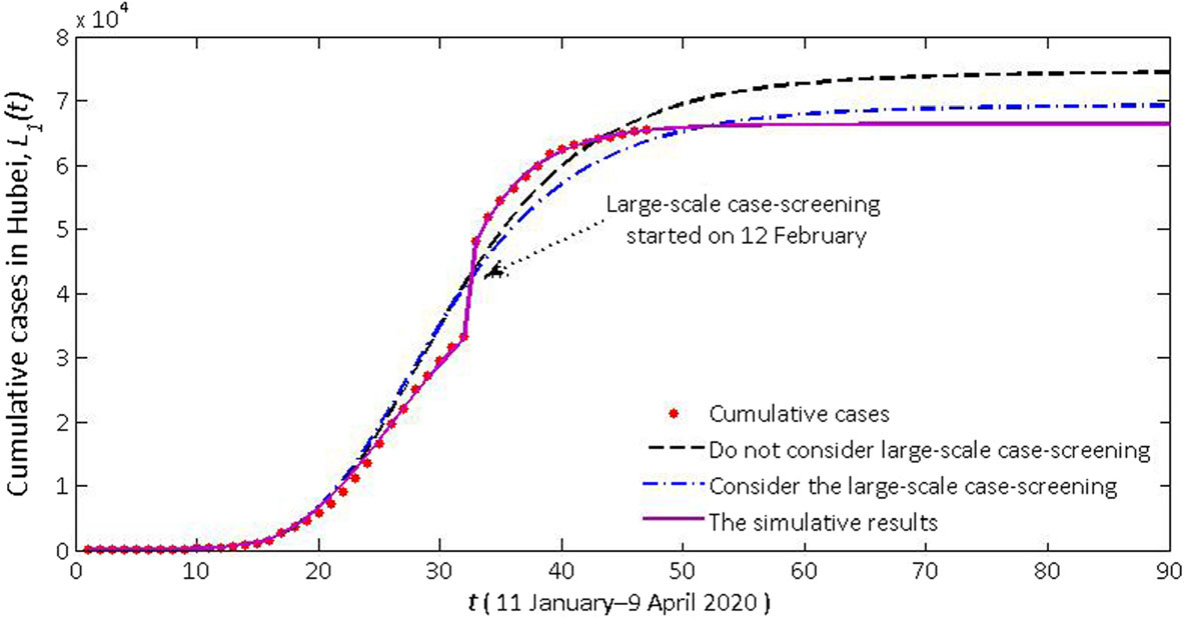
Impact of the large-scale case-screening (especially in Hubei province) on the number of predicted confirmed cases in Hubei province

**Fig. 5:**
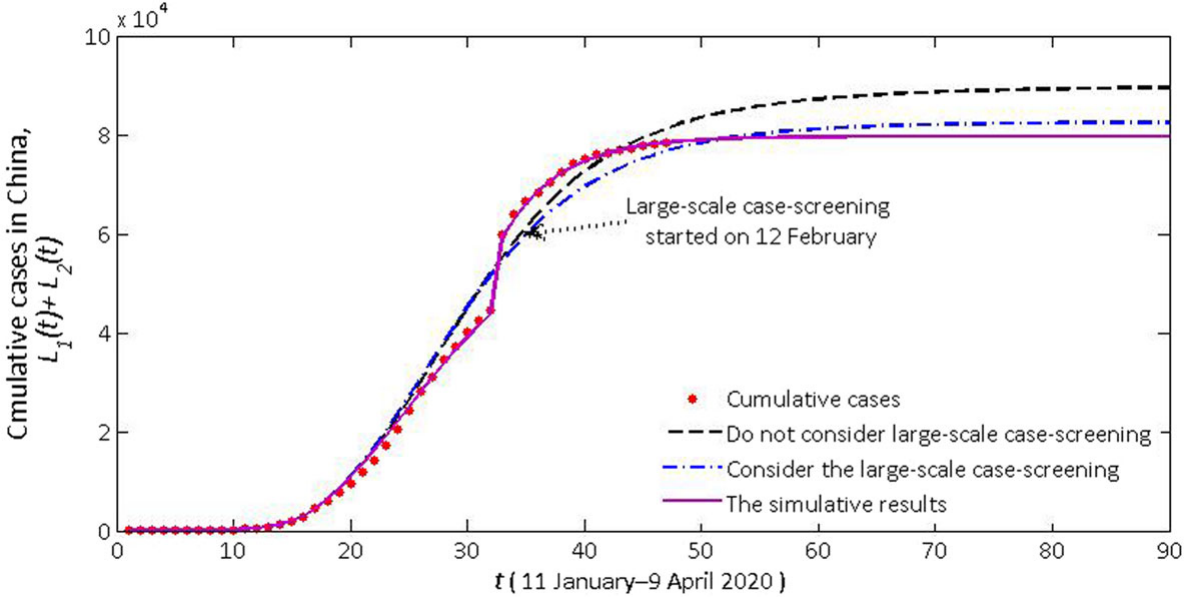
The number of predicted confirmed cases after the large-scale case-screening started on 12 February (especially in Hubei province) in the mainland of China

1) **If Wuhan was not locked down on 23 January, and no subsequent controlling measures were taken**: We use the parameters of the first stage (11 January–22 January 2020) and the initial values of the model to fit the cumulative confirmed case data of Hubei province (*L*_1_(*t*)) and the mainland of China (*L*_1_(*t*) + *L*_2_(*t*)) (Fig. 3). Cumulative cases in Hubei province will rapidly exceed 2 million within one month. So, it is clear that Wuhan was locked down on 23 January is very influential. After 23 January, the transmission rates (*β*_1_ and *β*_2_), migration rates (*ω*) and infectivity reduction factor (*l*) of the model (1) changed, but the quantity of each subgroup of the model (1) did not.

2) **If Wuhan was locked down on 23 January, but other cities in the mainland of China did not take controlling efforts**: We use the parameters of the first and second stage (11 January–26 January 2020). The initial values of the model to fit the cumulative confirmed case data of Hubei province (*L*_1_(t)) and the mainland of China (*L*_1_(t) + *L*_2_(t)) are seen in Fig. 3. Cumulative cases in Hubei province will be close to 1 million within one month. It is worth mentioning that with the lockdown of Wuhan, the transmission rate in overall Hubei has not decreased. However, the lockdown of Hubei is also workable.

3) **After Wuhan and Hubei were locked down, other cities in China were also locked down one after another, but the large-scale case-screening after 12 February was not initiated**: Since 12 February, NHC decided to add clinically diagnosed cases to confirmed cases, resulting in a “dramatic changes” after 12 February. Since 27 January (after Hubei is completely locked down), the clinically diagnosed cases, instead of the total number within the past 16 days, was reported by NHC every day. We assume that the model switches its pattern on 27 January, then we can get the cumulative confirmed cases of Hubei province and the mainland of China (black dotted line) in Figs. 4 and 5 during a time when large-scale case-screening was not conducted. It can be seen that the experimental numbers are greater than the actual numbers. And we see that due to the large-scale case-screening on 12 February, the system switches its pattern again. The cumulative confirmed cases of Hubei province and the mainland of China (blue dotted line) are shown in Figs. 4 and 5. Based on this conjecture, the cumulative number of confirmed cases nationwide was 82 186, and 69 230 in Hubei province on 9 April. This scenario validates the necessity of large-scale case-screening.

4) **Wuhan locked down, Hubei locked down, large-scale case-screening taken**: After Hubei province was locked down, we estimated that *S*_1_(17) = 3.25 × 10^4^, *S*_2_(17) = 1.02 × 10^4^. After the initiation of large-scale case-screening, we estimated that *S*_1_(33) = 1.94 × 10^3^, *E*_1_(33) = 1.83 × 10^3^, *I*_1_(33) = 4.24 × 10^3^, *Q*_1_(33) = 6.18 × 10^4^, *R*_1_(33) = 6.55 × 10^4^. Fitted with Matlab (solid purple lines), we estimated the real cases of Hubei province and the mainland of China (Figs. 4 and 5). The cumulative number of confirmed cases nationwide will be 79 633, and 66 386 in Hubei province in early March. If the current controlling measures are observed, the cumulative number of cases will gradually reduce in the future.

### Results of basic reproduction numbers

Next, we described the value of the basic reproduction number. From model (1), before Wuhan was locked down (before 23 January), *R*_0_ = 5.6015 in the mainland of China; after Wuhan was locked down and before Hubei was locked down (23–26 January), *R*_0_ = 6.6037. After Hubei province was locked down and large-scale case-screening was not started (27 January–11 February), the model (1) was divided into two independent models: *R*_0_^(1)^ = 3.7732 in Hubei; *R*_0_^(2)^ = 0.9943 outside Hubei province. After the large-scale case-screening, *R*_0_^(1)^ = 0.2020 in Hubei; *R*_0_^(2)^ = 0.0472 outside Hubei province. We could calculate the basic reproduction number in Hubei province on average as (5.6015 × 12 + 6.6037 × 4 + 3.7732 × 16 + 0.2020 × 14)/46 = 3.4094 until 25 February.

### Sensitivity analysis of basic reproduction number

**Fig. 6:**
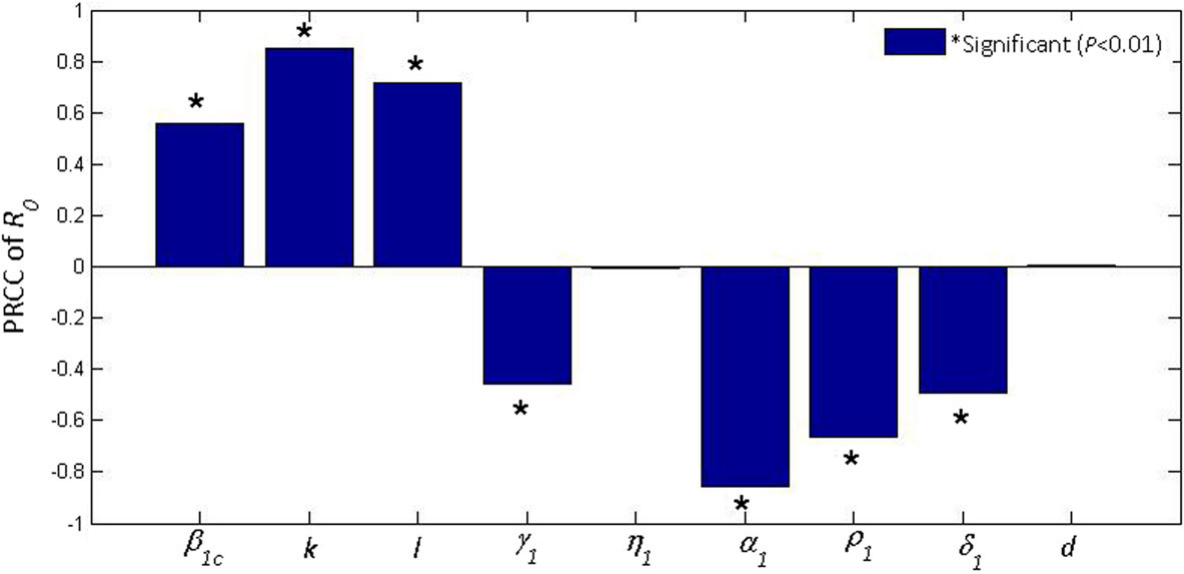
The values of (PRCC) on the outcome of *R*_0_^(1)^. All parameter values were derived from 27 January to 11 February 2020

In order to compare the sensitivity of these parameters to the basic reproduction number in Hubei (*R*_0_^(1)^) from 27 January to 11 February 2020, we calculated partial rank correlation coefficients (PRCC) with Latin Hypercube Sampling (LHS) [54] to detect the influence of each parameter with uncertain value on *R*_0_^(1)^. The sample size was chosen as *n* = 2000. We assumed the input parameters were in normal distributions. The expectations (i.e., parameter values) and standard deviations in Table 1. The significance level was chosen as 0.01. The partial rank correlation coefficients of *R*_0_^(1)^ were computed (Table 2). Figure 6 demonstrated its bar chart.

**Table 2:** Partial rank correlation coefficients (PRCC) values for *R*_0_^(1)^

Input parameter	PRCC	*P*-value
*α*_1_	−0.864972677	0
*k*	0.860166592	0
*l*_*c*_	0.734582947	0
*ρ*_1_	−0.658402698	0
*β*_1*c*_	0.550790936	0
*δ*_1_	−0.50823884	0
*γ*_1_	−0.460646049	0
*d*	0.043493277	0.052272305
*η*_1_	0.004758291	0.831920484

Particularly, the lager absolute value of the PRCC implies greater influence of certain parameter on the change of the cases newly infected with SARS-CoV-2. Thus it could be found that parameters *k*, *l*_*c*_, *β*_1*c*_, *d* and *η*_1_ had positive impacts on *R*_0_^(1)^; *α*_1_, *ρ*_1_, *δ*_1_ and *γ*_1_ had negative impacts. The sensitivity analysis showed that the basic reproduction number was highly sensitive to *α*_1_, *k*, *l*_*c*_, *ρ*_1_, *β*_1*c*_, *δ*_1_ and *γ*_1_. Therefore, lower transmission rate (*β*), lower infectivity reduction factor (*k* and *l*), shorter course of disease (1/*δ*) and higher detection rate (1/*γ*) could effectively reduce the basic reproduction number.

## Discussion

After the lockdown of Hubei province (around 27 January) and the large-scale case-screening (around 12 February), the transmission rate inside and outside Hubei province decreased significantly. In addition, since the lockdown of Hubei province, the basic reproduction numbers decreased significantly, indicating that the lockdown and large-scale case-screening are effective in controlling the epidemic rampancy across China.

After 27 January, in none-Hubei provinces, the basic reproduction numbers are almost less than one. Under the current conditions, the epidemics outside Hubei province are eventually be controlled, meaning that the cases outside Hubei province are mainly imported. Similarly, the epidemic situation in Hubei province has been basically controlled before and after the start of the large-scale case-screening. From Table 3, *R*_01_ > *R*_02_, *R*_03_ > *R*_02_ is seen in Hubei province, and *R*_04_ > *R*_05_, *R*_06_ > *R*_05_ in the other provinces, showing that close contact between susceptible people (*S*) and incubation patients (*E*), and medical staff (*S*) and isolated patients (*Q*) is the main route of transmission. The contact between susceptible individuals and suspected, carrier or undetected individuals in model (1) (uniformly defined as infected but not hospitalized individuals with asymptomatic transmission) is not the main route of transmission. Although the transmission rate between susceptible individuals and non-hospitalized patients is the biggest than that in *E* and *Q* classes, and the incubation period (1/*α*, average: 5.8 days) and treatment period (1/*δ*, average: 16.18 days) are longer than the detection time (1/*γ*, average: 4.41 days). This may be explained by the larger number of exposed patients (*E*) than those who are not hospitalized (*I*).

**Table 3:** The basic reproduction numbers of COVID-19 model (1)

11–22 January	23–26 January	27 January– 11 February	After 12 February
*R*_01_ = 1.8805	*R*_01_ = 2.5900	*R*_01_ = 2.0999	*R*_01_ = 0.1594
*R*_02_ = 0.5619	*R*_02_ = 0.7752	*R*_02_ = 0.6366	*R*_02_ = 0.0470
*R*_03_ = 3.1592	*R*_03_ = 3.2385	*R*_03_ = 1.0368	*R*_03_ = 1.3 × 10^−4^
*R*_04_ = 0.0891	*R*_04_ = 0.0466	*R*_04_ = 0.5534	*R*_04_ = 0.0362
*R*_05_ = 0.0434	*R*_05_ = 0.0240	*R*_05_ = 0.1677	*R*_05_ = 0.0110
*R*_06_ = 2.4444	*R*_06_ = 1.0353	*R*_06_ = 0.2732	*R*_06_ = 3.1 × 10^−5^
*R*_0_^(1)^ = 5.6015	*R*_0_^(1)^ = 6.6037	*R*_0_^(1)^ = 3.7732	*R*_0_^(1)^ = 0.2020
*R*_0_^(2)^ = 2.5697	*R*_0_^(2)^ = 1.1067	*R*_0_^(2)^ = 0.9943	*R*_0_^(2)^ = 0.0472
*R*_0_ = 5.6015	*R*_0_ = 6.6037	–	–

From Table 3, in the prophase of epidemic (11 January–26 January), the basic reproduction numbers in the mainland of China (*R*_0_) were 5.6015 and 6.6037. These results kept consistent with those of Tang et al. (6.47 [95% *CI*: 5.71–7.23]) [33], Shen et al., (4.71 [95% *CI*: 4.50–4.92]) [34] and Jia et al. (5.6870 inside Hubei, 6.0295 outside Hubei) [32]. In the metaphase of the epidemic (27 January–11 February), the basic reproduction number in Hubei was 3.7732, the average basic reproduction number was 3.4094 until 25 February. These results agreed with those of Zhao et al. (3.58 [95% *CI*: 2.89–4.39]) [35], Imai (1.5–3.5) [55], Read et al. (3.11 [95% *CI*: 2.39–4.13]) [56], Cao (4.08) [36], Abbott et al. (2.8–3.8) [37], Bedfordet et al. (1.8–3.5) [19], Chen et al. (2.30 from reservoir to person and 3.58 from person to person) [38] and Huang et al. (3.04–4.35) [39]. More research results argued that the basic reproduction number was less than 3, such as Liu et al. (2.92 [95% *CI*: 2.28–3.67]) [15], Li et al. (2.2 [95% *CI*: 1.4–3.9]) [18], Riou (2.2) [40], Majumder et al. (2.55) [41], Tang et al. (1.48–1.69 in Xi’an) [42], Zhang et al. (2.28 [95% *CI*: 2.06–2.52]) [57], Du et al. (1.32 [95% *CI*: 1.16–1.48]) [43] and so on. In fact, the basic reproduction number is closely related to time and region, and can reflect the severity of the epidemic.

In particular, the number of basic reproduction number in Hubei province was larger than that outside Hubei province. The number of basic reproduction number before the start of large-scale case-screening (prophase and metaphase of the epidemic) was much greater than that after the large-scale case-screening (anaphase of the epidemic). These results were consistent with the conclusion of Jia et al. [32], and this suggested that the epidemic in Hubei province was much more serious.

It also could be seen that our estimated basic reproduction number before 11 February 2020 was slightly higher than that of some previous studies [15, 18, 41–43, 57]. This might be mainly caused by the following two reasons. (1) When only Wuhan had confirmed cases at the prophase of the epidemic, we assumed that the number of susceptible individuals on 11 January was the permanent population of Wuhan. With the frequent migration of the population, the epidemic gradually spread across Hubei and China, so there was an increase in the number of susceptible persons before the lockdown of Hubei. But to simplify the discussion of the model, we have omitted this detail. (2) In addition, due to the existence a certain number of asymptomatic infections, the actual number of infections would exceed the confirmed case number released by NHC.

**Fig. 7:**
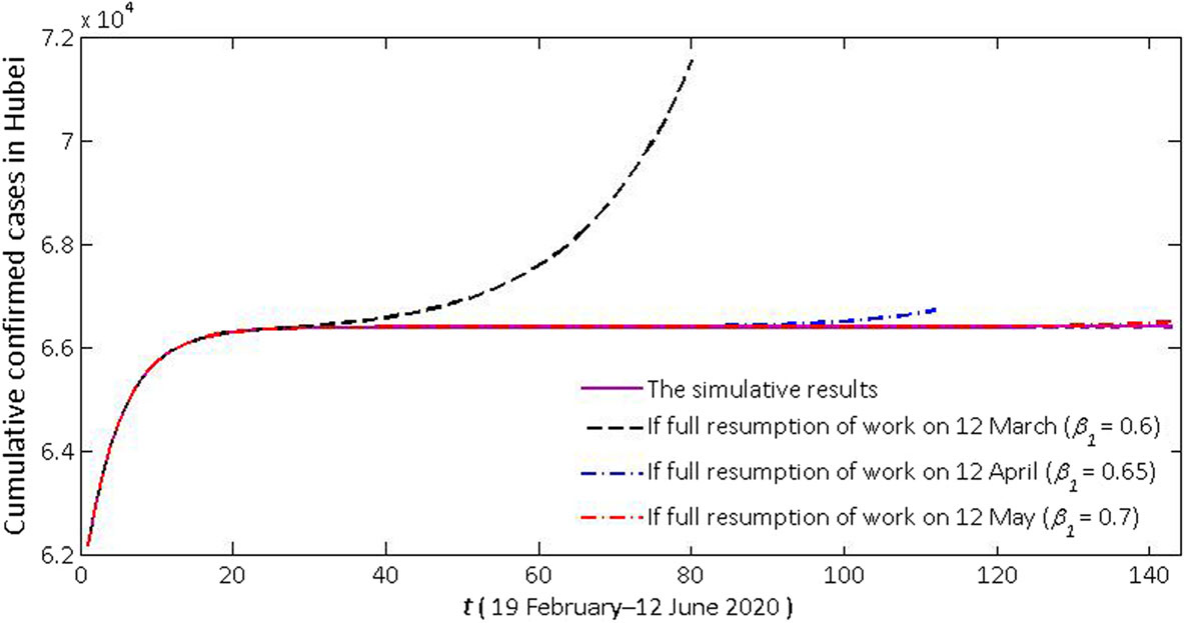
Effect of future migration (12 March, 12 April or 12 May) on the number of confirmed cases

We predicted the impact of the future migration in Hubei province on the epidemic status (see Fig. 7). Once the migration restarts, an increase will be observed in the number of susceptible persons and the transmission rate (*β*_1_). We assume that the number of susceptible persons in Hubei will mutate to 10 million. Next, we predicted the cumulative confirmed case at three time points (12 March, 12 April, and 12 May). We only predicted cumulative confirmed case data in the next 2 months. The transmission rates (*β*_1_) will be 0.6, 0.65 and 0.7. It is clear that even with low-level migration (only one million people are susceptible), protective efforts are still needed (the transmission rate is lower than the value of model (1) between January 11 and February 12). Once the migration starts on 12 March (*R*_0_^(1)^ = 1.4981), the epidemic situation will rise rapidly. If not controlled, the cumulative number of confirmed diagnoses in Hubei province after two natural months will exceed 71 500. If the migration starts on 12 April (*R*_0_^(1)^ = 1.6229) or 12 May (*R*_0_^(1)^ = 1.7477), even if the personal protection is slightly loose (the transmission rate increases in order), the epidemic seriousness will change relatively little within the next two natural months. Therefore, the current controlling efforts should not be eliminated too earlier. After the epidemic rampancy is completely controlled, theoretically, as long as sufficient precautions are taken (the transmission rate *β*_1_ is less than 0.4005 and the basic reproduction number is just less than 1), the epidemic situation will not break out again.

We obtained the research results on 25 February 2020, a day on which the epidemic in China was not completely quelled, and the global pandemic had not taken shape. At this stage, people cared about the time of the inflection point, the final number of cumulative confirmed cases, the time when the epidemic ended and normal daily activity resumed. In the current global pandemic, China is facing up with imported cases, asymptomatic infections, reinfection of confirmed patients and so on. The Chinese government has issued a series of countering measures, but we have not evaluated them and their impacts on future COVID-19 control, which will be our new research topic.

## Conclusions

China has curbed the spread of COVID-19 epidemic. Hubei province was the worst-hit area in China, especially its Wuhan. The lockdown of Hubei province resulted in a significant reduction in the basic reproduction number. The large-scale case-screening also shows the effectiveness in the epidemic control. The restart of population migration may bring with a risk of second outbreak. This shows that COVID-19 can be fundamentally controlled till its extinction. Although the epidemic is subsiding in China, the controlling efforts should not be terminated before May. This might provide experiences that can be replicated by other countries suffering from the pandemic.

## Data Availability

The data that support the findings of this study are available from the National Health Commission (NHC) (http://www.nhc.gov.cn/xcs/yqtb/list_gzbd.shtml), these network direct data are completely open, and we count these data day by day.

## Abbreviations

WHO: World Health Organization
COVID-19: Coronavirus disease 2019
SARS-CoV-2: Severe acute respiratory syndrome coronavirus 2
NHC: National Health Commission
GMT: Greenwich Mean Time
SARS-CoV: Severe acute respiratory syndrome coronavirus
MERS-CoV: Middle East respiratory syndrome coronavirus
SEIQR: Susceptible-exposed-infected but not hospitalized-infectious and isolated-recovered
CT: Computed tomography
